# CRP Is a Key Indicator of Rheumatoid Arthritis-Associated Vascular Injury and Neurodegeneration

**DOI:** 10.3390/ijms27115001

**Published:** 2026-05-31

**Authors:** Andreea Lazarut-Nistor, Melania Sibianu, Mark Slevin

**Affiliations:** 1 Doctoral School of Medicine and Pharmacy, George Emil Palade University of Medicine, Pharmacy, Science, and Technology of Targu Mures, 38th Gh. Marinescu Street, 540139 Targu Mures, Romania; andreealazarut@yahoo.com (A.L.-N.); melania.sibianu@gmail.com (M.S.); 2 Centre for Advanced Medical and Pharmaceutical Research, George Emil Palade University of Medicine, Pharmacy, Science, and Technology of Targu Mures, 38th Gh. Marinescu Street, 540139 Targu Mures, Romania

**Keywords:** inflammation, monomeric CRP, rheumatoid arthritis, vascular injury, neurodegeneration

## Abstract

Systemic inflammation plays a pivotal role in the progression of rheumatoid arthritis (RA) and its associated comorbidities, ranging from cardiovascular (CV) disease to neurodegenerative conditions such as Alzheimer’s disease (AD). This narrative review examines the molecular cross-talk linking these pathologies, with a specific focus on the distinction between pentameric C-reactive protein (pCRP) and its proinflammatory monomeric form (mCRP). We discuss evidence suggesting that mCRP is not merely a passive marker but also an active driver of endothelial dysfunction, atherosclerosis, and synovial inflammation. This review further explores the connections among inflammatory biomarkers, blood vessel integrity, and neurodegeneration, detailing how persistent cytokine elevation (IL-6, TNF-α) and vascular injury contribute to cerebral small vessel disease (cSVD) and cognitive decline, with neurofilament light chain (NfL) serving as a key biomarker of neuroaxonal injury. Additionally, we address the neurobiology of pain in RA, highlighting the mechanisms of central sensitization (CS) and neuroimmune signalling that sustain pain-independent joint swelling. This evidence indicates that understanding the dynamic connection between CRP isoforms and neuronal markers should offer new insights for risk stratification and suggests that targeting mCRP may provide a novel therapeutic avenue to mitigate both articular and extra-articular manifestations of RA.

## 1. Introduction

Systemic inflammation plays a pivotal role in the development and progression of many chronic diseases, ranging from autoimmune disorders such as rheumatoid arthritis (RA) to neurodegenerative conditions such as Alzheimer’s disease (AD). Among the most widely studied inflammatory biomarkers, C-reactive protein (CRP) has long served as a key clinical indicator of inflammatory activity. CRP is an acute-phase reactant synthesized predominantly by hepatocytes in response to IL-6 signalling, although extrahepatic expression has been documented in macrophages, endothelial cells, adipocytes, and neurons under conditions of systemic or localized inflammation. Its principal biological functions include complement pathway activation, opsonization of cellular debris, facilitation of apoptotic cell clearance, and modulation of innate immune effector mechanisms. In contrast to pCRP, mCRP has been shown to potentiate endothelial activation, promote leukocyte transmigration, and augment cytokine elaboration, implicating it as a functionally active mediator rather than a passive inflammatory correlate. However, recent evidence highlights the importance of distinguishing between pentameric CRP (pCRP) and its monomeric form (mCRP), as their relative abundance and ratio seem to have distinct biological and prognostic implications. A high CRP-to-mCRP ratio is typically observed in acute-phase inflammatory responses, whereas a low ratio characterizes chronic inflammatory states (for example, in RA), vascular injury, and neurodegenerative conditions, suggesting that shifts in CRP conformations can reflect the underlying inflammatory context.

In autoimmune diseases such as RA, persistent systemic inflammation significantly increases the risk of CV complications, which represent a leading cause of mortality among these patients. Multiple studies have demonstrated that certain key mediators of RA pathogenesis, such as CRP, IL-6, and TNF-α, are strongly associated with carotid intima–media thickness (cIMT), an established marker of subclinical atherosclerosis. Both IL-6 and TNF-α contribute to endothelial dysfunction and vascular injury, thereby accelerating atherogenesis. Moreover, anticitrullinated peptide antibodies (ACPAs), a hallmark of RA, have been linked to increased CV risk independent of traditional risk factors. The cumulative inflammatory burden, as reflected by disease activity scores such as DAS28-CRP, further amplifies the likelihood of subclinical atherosclerosis, highlighting the chronic inflammatory state as a key driver of vascular pathology in RA [1].

Longitudinal and cross-sectional studies have consistently reported structural and functional vascular alterations in RA. In cohorts without CV disease, flow-mediated endothelial dysfunction and increased cIMT correlate with disease duration, indicating progressive vascular compromise over time. Early in the disease course, CRP levels appear to predict future vascular changes, with elevated inflammatory markers at diagnosis associated with accelerated atherosclerotic progression. Notably, while some studies reported no association between the erythrocyte sedimentation rate (ESR) and cIMT, CRP demonstrated a robust correlation, reinforcing its role as a more sensitive marker of vascular inflammation in RA [2,3,4].

In addition to autoimmune and vascular contexts, chronic systemic inflammation also contributes to neurodegenerative processes. Persistent elevation of inflammatory mediators, particularly CRP and IL-6, is associated with neuronal injury and is reflected in increased concentrations of neurofilament light chain (NfL) in the cerebrospinal fluid (CSF) and plasma. NfL, a structural component of the neuronal cytoskeleton, is released into the circulation following axonal damage and is increasingly recognized as a sensitive biomarker for neurodegeneration. Recent studies have proposed that NfL may serve as a “neurological CRP,” reflecting the degree of neuronal stress or injury analogous to how CRP mirrors systemic inflammation [5]. Accumulating evidence indicates that pCRP does not readily traverse an intact BBB, yet mCRP has been identified within the cerebral vasculature and parenchymal tissue, particularly in the context of BBB compromise. The neuroinflammatory sequelae attributable to mCRP operate through an indirect pathway involving endothelial activation and disruption of barrier integrity, resulting in increased BBB permeability, and a direct pathway characterized by microglial polarization toward a proinflammatory phenotype accompanied by increased cytokine secretion [6].

Pain in RA also has an impact systemically and is not only influenced by joint inflammation but also involves other complex neurobiological processes. Peripheral sensitization occurs when proinflammatory cytokines, particularly TNF-α, IL-1β, IL-6, and IL-17, activate and sensitize nociceptors in the joints, amplifying pain signals. Moreover, central pain mechanisms play a key role, with microglia and astrocyte activation in the spinal cord contributing to increased excitatory neurotransmission through mediators such as glutamate and calcitonin gene-related peptide (CGRP) and reduced inhibitory control within the central nervous system. These processes help maintain pain even when inflammation is controlled, explaining why many RA patients continue to experience pain despite effective anti-inflammatory therapy [7].

Chronic pain is also closely linked to low-grade inflammation, further illustrating the interaction between CRP elevation, amyloid metabolism, and neurodegenerative risk. Elevated CRP levels in individuals with chronic pain correlate with increased plasma concentrations of amyloid-β40 and amyloid-β42, peptides implicated in AD pathogenesis. This inflammatory state is also associated with smaller hippocampal volumes and greater risk of cognitive decline. Similarly, population-based studies in older adults have revealed positive correlations between the levels of inflammatory cytokines (IL-6, TNF-α, and IL-8) and the levels of neurodegeneration markers such as NfL, emphasizing the systemic-to-neural chain of inflammation [8,9].

Hence, persistent inflammation links autoimmune, vascular, and neurodegenerative diseases through shared molecular mediators and pathophysiological mechanisms. In RA, chronic inflammation not only furthers joint destruction but also contributes to persistent pain, which reflects ongoing immune-mediated tissue damage and central sensitization (CS). Understanding the dynamic interconnection between CRP isoforms, particularly mCRP, cytokines, and neuronal injury markers such as NfLs will provide critical insights into the common inflammatory pathways underlying these conditions and open new possibilities for targeted therapeutic interventions.

The purpose of this narrative review is to highlight novel signalling pathways and mechanisms that describe how systemic inflammation, reflected through the interaction between CRP/mCRP isoforms and proinflammatory/injury markers such as NfL, connects autoimmune, vascular, and neurodegenerative diseases, thereby clarifying shared pathogenic pathways and potential new therapeutic targets.

## 2. RA—A Chronic Systemic Immune-Mediated Inflammatory Disease

### 2.1. TNF-α/IL-6 Pathways and the Amplification of Proinflammatory Signalling in RA

RA is a systemic inflammatory condition characterized by a dysregulated immune response that involves autoantibodies, rheumatoid factor (RF) and ACPAs, which form immune complexes that activate macrophages and other immune cells, leading to elevated levels of proinflammatory cytokines (especially TNF-α and IL-6), which increase synovial inflammation, cartilage destruction and bone erosion [10,11]. The inflammation associated with RA places patients at increased risk of vascular disease, even when joint symptoms are managed, with inflammation persisting and manifesting in extra-articular tissues (lungs, heart, vessels) [12]. Several signalling pathways have been shown to play key roles in disease progression: the JAK-STAT pathway promotes FLS survival and proinflammatory immune responses, the MAPK pathway regulates cytokine production and tissue degradation, and the NF-κB pathway amplifies inflammatory signalling and bone erosion through feedback mechanisms [13].

Clinical studies with TNF inhibitors (e.g., adalimumab, infliximab, and etanercept) demonstrated that blocking TNF-α suppressed downstream cytokine cascades, reduced CRP levels, halted joint damage, and improved systemic symptoms [14].

### 2.2. IL-6 Is a Key Mediator Linking Local Joint Inflammation to CRP Involvement

RA has been linked to multiple severe comorbidities that are caused by proinflammatory cytokines that maintain systemic inflammation, with IL-6 being the main cytokine produced by synovial fibroblasts, macrophages, endothelial cells, T cells, B cells, and other stromal elements in the inflamed joint. Specifically, IL-6 induces myocardial and vascular dysfunction, leading to left ventricular fibrosis in CV disease, directly correlates with insulin resistance in metabolic syndrome, and disrupts bone homeostasis by promoting bone resorption while inhibiting bone formation in osteoporosis. To combat these widespread effects, several therapeutic strategies specifically target the IL-6 pathway; for example, the IL-6 receptor antagonist tocilizumab offers antifibrotic benefits for RA-associated interstitial lung disease and rapidly improves insulin sensitivity, and the JAK inhibitor tofacitinib prevents joint damage and bone loss by inhibiting IL-6 secretion [15]. IL-6 also has endocrine-like effects beyond joint tissues, influencing haematopoiesis (e.g., anaemia in chronic disease), metabolic regulation, and neuroimmune cross-talk. Clinical IL-6 blockade (e.g., tocilizumab) strongly suppresses CRP synthesis and systemic inflammation, emphasizing the central regulatory role of IL-6 and its close relationship with CRP, which is the main subject of this review [13].

In RA, IL-6 signalling occurs through two pathways: classic signalling and trans-signalling. Classic signalling involves the binding of IL-6 to the membrane-bound IL-6 receptor on a limited number of cell types, which mainly mediates protective and regenerative effects. In contrast, trans-signalling occurs when IL-6 binds to the soluble IL-6 receptor, allowing the activation of gp130 on many cell types, including synovial and endothelial cells. In RA, IL-6 trans-signalling is particularly important in promoting chronic inflammation, leukocyte recruitment, and joint damage. Importantly, although modulating IL-6 pathways (either directly or indirectly) can significantly reduce inflammatory cytokine production, angiogenesis, and tissue damage, studies in IL-6-deficient mice revealed that complete loss of IL-6 impaired protective regenerative functions, particularly in the intestine, where epithelial repair depends on classic signalling via the membrane-bound IL-6 receptor. IL-6 trans-signalling mediates the proinflammatory effects of IL-6, whereas classic signalling supports anti-inflammatory and regenerative processes, suggesting that selective trans-signalling blockade is therapeutically superior to global IL-6 inhibition [16].

### 2.3. CRP Functions as Both a Biomarker and a Potential Effector of Inflammation in RA

**Figure 1:**
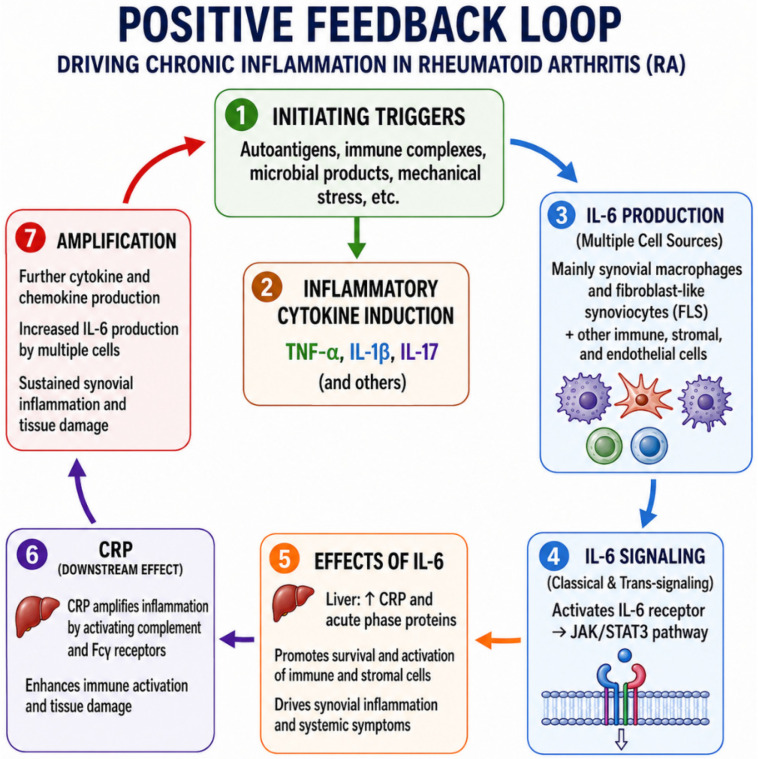
Molecular and cellular mechanisms underlying the positive feedback loop in rheumatoid arthritis (RA). This schematic illustrates the positive inflammatory feedback loop involved in rheumatoid arthritis (RA). Initial inflammatory triggers, including autoantigens, immune complexes, microbial products, and mechanical stress, promote the induction of the proinflammatory cytokines TNF-α, IL-1β, and IL-17. These mediators stimulate IL-6 production primarily by synovial macrophages and fibroblast-like synoviocytes (FLSs), with additional contributions from T cells, B cells, dendritic cells, neutrophils, osteoclasts, adipocytes, and endothelial cells. IL-6 signalling through classical and trans-signalling pathways activates downstream JAK/STAT3 signalling, promoting synovial inflammation, angiogenesis, osteoclastogenesis, and systemic inflammatory responses [13]. IL-6 also stimulates the hepatic synthesis of C-reactive protein (CRP) and other acute-phase proteins. CRP further amplifies inflammation through complement activation, Fcγ receptor-mediated immune activation, and increased cytokine and chemokine production, thereby sustaining the chronic inflammatory cycle characteristic of RA [18]. The continuous interaction between TNF-α, IL-6, CRP, and downstream cytokine pathways perpetuates chronic inflammation in RA. (Generated by Google Gemini 3 Pro).

CRP is synthesized in the liver in response to IL-6 (and, to a lesser extent, IL-1β and other cytokines). In RA, CRP is a solid biomarker of systemic inflammation and is used clinically to monitor disease activity, guide therapy, and predict radiographic progression. Elevated CRP levels often parallel disease flares and decrease with effective biologic therapy, particularly IL-6 or TNF blockers, as reviewed by Pope et al. [17]. Recent data suggest that CRP (especially its monomeric form) may not be completely passive, as it can activate complement pathways, engage Fc receptors, and promote local inflammatory amplification in tissues, acting as both a marker and a modulator of inflammation (discussed in detail later) [18]. The exposure of neoepitopes in mCRP allows for the targeted detection of tissue-bound CRP via specific antibodies. Crucially, the transition from pentameric to monomeric CRP “unlocks” the C1q binding site, a necessary step for activating the complement system (see Figure 1). In the context of RA, inflammatory surges increase CRP production, and depending on its structural state, this abundance of CRP likely fuels disease progression by activating the complement cascade and altering cellular signalling [19].

CRP inhibited bone remodelling in RAW 264.7 mouse monocyte/macrophage lines treated with oxPAPC, a phospholipid known to downregulate Toll-like receptors (TLRs), and disrupted the MAPK signalling pathway, which reduced osteoclast formation, decreased key bone-building transcription and ultimately prevented normal bone mineralization. These effects are mediated through TLR signalling; when these receptors are blocked, the inhibitory effects of CRP are reversed. Elevated CRP levels do not simply indicate inflammation but actively contribute to a low degree of bone turnover, providing a molecular explanation for the bone loss and increased fracture risk observed in patients with chronic inflammatory conditions such as RA. When the cells were treated with oxPAPC before the introduction of CRP, a “rescue” effect was observed: the suppression of osteoclast differentiation was reversed, and inflammatory signalling (specifically the TRIF pathway) was neutralized. This study suggested that CRP docked at TLRs to send inhibitory signals. Specifically, oxPAPC acted as a shield and blocked the receptor for CRP, losing the ability to disrupt the bone remodelling process [20].

Compared with those from healthy controls, RA-FLSs from the synovial tissue of 21 RA patients significantly overexpressed the CRP receptors CD32 and CD64. Further in vitro studies using FLSs derived from patient tissue revealed that when CRP bound to these receptors, increased cell proliferation and tissue invasion were detected, and the proinflammatory cytokines IL-6 and CXCL8 were concomitantly released. CRP acts through two distinct signalling routes: a CD32-driven NF-κB pathway that controls inflammation and a CD64-driven p38 MAPK pathway that regulates tissue-destroying enzymes via MMP9. In addition, the expression of the anti-inflammatory cytokine IL-10 was suppressed following CRP stimulation, further shifting the joint environment toward chronic destruction. Therefore, this evidence strongly suggests that CRP is a direct pathogenic driver of synovial inflammation rather than just a byproduct [21].

## 3. Vascular Damage in RA Occurs Primarily as a Consequence of Chronic Systemic Inflammation

### 3.1. Microvascular Injury, Arterial Inflammation, and Structural Remodelling in RA

Chronic inflammation in RA causes endothelial activation and dysfunction, which eventually leads to atherogenesis. There is reduced endothelial nitric oxide bioavailability, increased expression of adhesion molecules (VCAM-1, ICAM-1), increased circulating asymmetric dimethylarginine (ADMA), and oxidative stress in RA patients, all of which are linked to impaired vasodilatory responses and a prothrombotic endothelial phenotype. Endothelial dysfunction in the RA has been shown both in conduit arteries (flow-mediated dilation studies) and in the microcirculation [22].

Capillary rarefaction, abnormal capillary morphology, and impaired microvascular reactivity were observed in RA patients early in the disease course with the use of nailfold video capillaroscopy and other microcirculation assessments. These changes are correlated with inflammatory markers and disease activity, suggesting that chronic synovial inflammation furthered with systemic endothelial activation that led to microvascular injury and dysfunctional angiogenesis and accelerated arterial remodelling and atherogenesis [23]. Imaging studies revealed increased arterial wall inflammation (measured by 18F-FDG PET) and increased arterial stiffness in RA patients, indicating that both the functional (endothelial) and structural (arterial wall remodelling and fibrosis) compartments were affected. In a cross-sectional study of patients aged ≥50 years, arterial FDG uptake was assessed in early untreated RA patients, established RA patients, and age- and sex-matched osteoarthritis (OA) controls. RA patients showed significantly greater FDG uptake in the aorta, carotid, and femoral arteries than OA patients did, even after adjusting for traditional CV risk factors. Early RA patients presented the highest arterial FDG uptake, followed by established RA and OA patients. Arterial FDG uptake in RA patients was strongly associated with markers of systemic inflammation (ESR, CRP, and therefore the DAS28 score), whereas traditional CV risk factors showed limited associations. Adjustment for inflammatory markers attenuated the differences between RA and OA, indicating that systemic inflammation partly explains the increased vascular inflammation [24].

Detailed mechanistic information was provided by Eisenhardt et al., who reported that within human aortic and carotid atherosclerotic plaques, CRP was present almost exclusively in its monomeric form, not as circulating pCRP. Activated platelets and apoptotic cells rapidly and completely dissociated pCRP into mCRP through a membrane lipid-dependent mechanism mediated by lysophosphatidylcholine (LPC), with native polyacrylamide gel electrophoresis used to assess pCRP dissociation. This conversion occurs locally on cell membranes, including in whole blood, and mCRP remains bound to the membrane surface. Functionally, mCRP was more proinflammatory than pCRP at low, clinically relevant concentrations and activated monocytes, increasing Mac-1 integrin activation, reactive oxygen species production, and adhesion to fibrinogen, which are key steps in leukocyte recruitment, adhesion, and transmigration during atherogenesis. These effects depend largely on lipid rafts and Fcγ receptors and involve the PI3K and src-kinase signalling pathways. In contrast, pCRP showed weak proinflammatory activity at low concentrations and partially inhibited the effects of mCRP. These findings demonstrated that the proatherogenic effects of CRP are caused primarily by its local dissociation into membrane-bound mCRP, which has potent proinflammatory effects, whereas circulating pCRP has comparatively weak activity [25].

A prospective study monitored 108 RA patients over 15 years to investigate the impact of early inflammation on vascular health (specifically, the development of arterial stiffness, a key independent risk factor for CV disease). The results identified baseline CRP levels as a robust predictor of future arterial stiffness, independent of conventional CV disease risk factors such as hypertension, age and sex. Arterial stiffness was noninvasively assessed via pulse-wave velocity (PWV), the gold standard for large-artery stiffness, and the Augmentation Index (AIx), both of which strongly predict baseline CRP; elevated initial CRP correlated with significantly greater odds of vascular impairment, with an AIx OR of 3.52 (95% CI 1.04–11.90) and a PWV OR of 4.84 (95% CI 1.39–16.83) at the 15-year follow-up, confirming that the chronic inflammation inherent to the RA is a direct contributor to structural arterial damage and endothelial dysfunction [26].

### 3.2. Endothelial Adhesion Molecules Are Strong Predictors and Early Indicators of Vascular Injury in RA

A comprehensive clinical study involving 50 long-standing RA patients, 50 newly diagnosed RA patients, and 50 controls evaluated serum VCAM-1 as a biomarker for endothelial dysfunction by correlating it with disease activity (DAS28), oxidative stress, and inflammatory markers under the premise that RA increases CV risk. The results revealed significantly higher serum VCAM-1 levels in RA patients than in controls, confirming the presence of endothelial dysfunction. Notably, VCAM-1 levels were higher in newly diagnosed patients than in treated long-standing patients, suggesting that effective disease control improved endothelial health. Furthermore, RA patients, especially those newly diagnosed with RA, presented elevated oxidative stress (high MDA and low antioxidant capacity) and inflammatory markers (CRP and ESR), and VCAM-1 was positively correlated with MDA and inflammatory markers and negatively correlated with antioxidant capacity, indicating that oxidative stress and inflammation serve as predictors of endothelial dysfunction and CV risk in RA patients [27].

In vivo mechanistic data from B6 mice susceptible to both arthritis and atherosclerosis revealed that CIA and a high-lipid diet (HD) exert distinct, independent effects on the vasculature. CIA causes early systemic vascular dysfunction, which is specifically characterized by the overexpression of the adhesion molecule VCAM-1 in the aorta. In contrast, when B6 mice were fed a high-lipid diet, atheroma plaque formation and increased iNOS expression were independently induced. The combination of CIA and HD did not amplify vascular damage, confirming that the proinflammatory environment of arthritis and metabolic lipid stress contributed to large vessel injury through separate pathways. While VCAM-1 expression in the aortic sinus is influenced by both factors, its overexpression in the aorta serves as a specific early marker for arthritis-induced inflammation [28].

## 4. CRP Isoforms and Neuroinflammatory Biomarkers Across Systemic and Tissue-Specific Inflammation

Monomeric CRP was first considered a contributor to the development of CV and cerebrovascular disease pathology through dysregulation and inflammation of the endothelium in the first decade of the 21st century, but its true value as both a predictor and promotor of tissue damage is only now becoming clear. When nCRP encounters damaged tissue, hypoxic environments, or cell membranes, it dissociates into mCRP, a more biologically active form. Unlike nCRP, mCRP is localized within vessel walls and diseased tissues, where it primarily promotes endothelial cell activation, inflammatory cytokine release, reactive oxygen species generation, complement activation, platelet adhesion, and thrombus growth. mCRP also stimulates angiogenesis by activating key intracellular signalling pathways such as ERK1/2, enhancing endothelial migration, tube formation, and neovessel development. Tissue studies have shown that mCRP accumulates in atherosclerotic plaques, peri-infarct brain and cardiac tissue, and amyloid-associated vessels in AD, particularly in areas rich in newly formed microvessels [29].

### 4.1. mCRP May Have an Active Effect on RA Pathogenesis

pCRP has different functions than its monomeric counterpart, with distinct biological properties. Recent findings have shown that mCRP is not just a passive indicator but also an active contributor to RA pathogenesis, as it accumulates within inflamed tissues [17]. Jia et al. were the first to define the conformation-specific role of mCRP in osteoclast differentiation and synovial biology in RA, particularly at the level of osteoclast precursors [30]. Endotoxin-free highly purified pCRP was converted into mCRP along with mutants lacking the membrane-binding cholesterol sequence. Functional comparisons revealed that mCRP, but not the native pentameric form, displayed a distinct capacity to bind RANKL. Global and microarray profiling revealed that mCRP significantly modulated intracellular signalling cascades downstream of RANKL, including NF-κB activation and phospholipase C-dependent pathways, and inhibited osteoclast differentiation in vitro, indicating a suppressive role in osteoclast formation and bone resorptive activity. Compared with control mice, CRP-deficient mice presented enhanced bone resorption, which was consistent with the loss of a protective regulatory influence normally mediated by CRP. Differentially expressed genes (11 out of 35) identified in response to mCRP stimulation were subsequently validated by quantitative PCR, confirming the strong regulation of osteoclast-associated and inflammatory transcripts [30].

Human primary chondrocytes (from OA patients and healthy donors) and the murine chondrocyte cell line ATDC5 exposed to mCRP in vitro presented robust and sustained proinflammatory and catabolic responses characterized by increased nitric oxide production and marked upregulation of inflammatory and cartilage-degrading mediators, including NOS2/iNOS, COX-2, MMP13, VCAM1, IL-6, IL-8, and LCN2, at both the mRNA and protein levels. mCRP triggered similar responses in chondrocytes derived from healthy donors. Pharmacological inhibition of NF-κB signalling with pyrrolidine dithiocarbamate (PDTC) significantly attenuated mCRP-induced mediator expression, confirming that NF-κB is a key pathway for mCRP activity in chondrocytes [31]. A similar study utilized FLSs from the RA synovium, which respond to mCRP through Fcγ receptors (CD32/CD64) and downstream activation of NF-κB and p38 MAPK, linking this mechanism to the chronic, self-sustaining nature of synovial inflammation [18].

This is the only evidence indicating that the monomeric form of CRP plays distinct and biologically active roles within the joint environment. Together, these findings suggest that mCRP is not merely an inflammatory marker but also an active modulator of RA pathogenesis, influencing osteoclast differentiation, synovial inflammation, and cartilage degradation through pathways such as the NF-κB pathway. However, further studies are needed to clarify its in vivo relevance and therapeutic potential.

### 4.2. The mCRP:pCRP Ratio as a Candidate Biomarker of Local Tissue Inflammation in RA

The two isoforms differ in their biological activity, with pCRP being less inflammatory or even regulatory in some settings, whereas mCRP displays potent proinflammatory effects in tissues. This structural and functional dichotomy provides the conceptual foundation for studying the mCRP/pCRP ratio as a marker of the balance between systemic CRP and locally generated, tissue-acting mCRP. Clinical research focusing on vasculitis has shown that circulating mCRP levels and the mCRP/pCRP ratio may be more sensitive indicators of inflammatory activity in RA patients and related arthritides than pCRP alone, although assay standardization remains a barrier to widespread clinical use [32].

A clinical study that directly measured conformation-specific mCRP revealed markedly higher absolute mCRP and mCRP/pCRP ratios in adult-onset Still’s disease (AOSD) patients than in RA patients, polymyalgia rheumatica patients or infection patients; RA patients presented elevated mCRP relative to healthy controls, but the mean mCRP/pCRP ratio in RA patients was substantially lower than that in AOSD patients [33]. These disease-specific patterns suggest that the ratio may discriminate inflammatory syndromes with differing degrees of local CRP conversion or tissue-level inflammation. Specifically, the data revealed that mCRP levels were higher in RA patients than in healthy controls. The mCRP:pCRP ratio in RA patients was generally lower than that in patients with hyperinflammatory syndromes such as AOSD but was still elevated in RA patients than in healthy subjects, and mCRP biology (osteoclast modulation, FLS activation) revealed plausible pathways by which a greater local mCRP burden contributes to joint erosion and synovitis. Thus, the ratio could have potential as a biomarker for local joint inflammation, a stratifier for patients who might benefit from mCRP-targeted approaches, or as a pharmacodynamic readout for drugs that prevent pCRP to mCRP conversion; however, larger RA-focused cohort studies with harmonized assays and synovial fluid/tissue correlation are still needed [33].

Additional in vitro studies using circulating microparticles (MPs) derived from cell cultures, from postacute MI patients and from controls revealed that MPs both generated and transported mCRP, whereas inhibitors of pCRP binding blocked that conversion. The mCRP:pCRP ratio is driven by local conversion processes: activated cell membranes, apoptotic cells, and MPs bind pCRP and promote its dissociation into mCRP, with local synthesis of CRP in inflamed tissues. Tissue mCRP was present from both local production and in situ dissociation of circulating pCRP. These mechanisms explain why an increased mCRP:pCRP ratio reflects active tissue inflammation even when systemic pCRP is modest [34].

However, translating these mechanistic insights into clinical practice depends critically on the ability to accurately distinguish and quantify CRP isoforms. Conformation-specific assays are needed for reliable measurement, since many standard high-sensitivity CRP tests detect pCRP only and cannot distinguish isoforms. Published studies used ELISAs or reagents (antibodies/aptamers) that specifically recognized mCRP; assay variability, preanalytic sample handling (e.g., freezing/thawing, platelet/microparticle removal), and differences in how the ratio was scaled (some reported mCRP in ng/mL and then expressed the ratio multiplied by a factor) complicate cross-study comparisons. Because of these technical issues, the mCRP:pCRP ratio is promising but has not yet been standardized for routine clinical use [31].

### 4.3. CRP and NfL Could Be Utilized as Context-Dependent Biomarkers of Inflammation and Tissue Injury

NfL, a structural neuronal protein released following axonal damage, serves as a sensitive biomarker of neuroaxonal injury across both acute inflammatory and chronic neurodegenerative conditions. In acute COVID-19, blood NfL concentrations increase in proportion to disease severity and are positively correlated with CRP, with inflammatory markers collectively predicting NfL levels, suggesting concurrent systemic inflammation and neuroaxonal injury. Notably, even patients with mild COVID-19 who lacked overt neurological manifestations presented significant correlations between serum NfL and CRP at baseline and during follow-up, supporting the presence of inflammation-linked, subclinical axonal injury. Similar patterns were observed in critical illness and prolonged systemic inflammation, where greater inflammatory burden during intensive care unit treatment was associated with higher NfL concentrations and subsequent cognitive impairment, which is consistent with parallel elevations of CRP and NfL under conditions of strong, active inflammatory stress. While CRP reflects the magnitude of systemic inflammatory activation, NfL more directly captures the downstream neuronal consequences of inflammation-related or degenerative tissue injury [35]. In contrast, the relationship between CRP and NfL is less consistent in chronic or neurodegenerative conditions such as AD, mild cognitive impairment (MCI), and aging. In these contexts, NfL levels primarily reflect ongoing neurodegeneration, whereas CRP levels variably represent the systemic inflammatory tone. Several studies reported that NfL correlated more robustly with specific proinflammatory cytokines than with CRP itself, suggesting that low-grade inflammation only modestly mediated axonal injury in chronic disease states. Accordingly, NfL has been described as the “CRP of neurology,” functioning as a broad biomarker of neuronal damage rather than one tightly coupled to systemic inflammation. Thus, while CRP and NfL correlated under conditions of pronounced acute inflammation, their associations weakened in chronic, mixed-aetiology settings, where they reflected partially distinct biological pathways [36]. Importantly, NfL, CRP, and mCRP represent fundamentally different levels of the pathological cascade and therefore provide complementary rather than interchangeable biological information. NfL is primarily a passive structural biomarker released into the circulation following neuroaxonal injury and reflects the extent of ongoing neuronal damage irrespective of the initiating cause. In contrast, native pentameric CRP largely functions as a systemic inflammatory biomarker produced by the liver in response to cytokine signalling, particularly IL-6, and reflects the magnitude of peripheral inflammatory activation. However, mCRP differs substantially from both molecules in that it acts not merely as a marker but also as a biologically active mediator capable of directly amplifying endothelial dysfunction, oxidative stress, complement activation, microglial activation, BBB disruption, and prothrombotic signalling within damaged tissues. Thus, while elevated NfL reflects the downstream consequence of neurodegeneration or neuroinflammation, mCRP may participate more proximally in driving pathological neurovascular and inflammatory processes that contribute to neuronal injury itself. Accordingly, NfL may be best interpreted as an indicator of accumulated neuroaxonal damage, whereas CRP, especially mCRP, may reflect upstream inflammatory mechanisms and active disease propagation.

Hence, while the mCRP isoform seems to be a key driver of tissue damage and neurodegenerative decline, NfL acts merely as a biomarker, albeit well, reflecting tissue damage; hence, together or as part of a panel, NfL may represent an AI-type algorithmic tool for predicting outcomes and responses to therapy.

## 5. Neurodegeneration and Cognitive Decline Are Strongly Associated with Chronic Systemic Inflammation

### 5.1. CRP and IL-6 as Complementary Biomarkers of Neurodegeneration

In recent years, biomarkers that reflect different pathological axes, such as inflammation, neuronal injury, and degeneration in AD, have become increasingly important, with CRP and IL-6 serving as systemic markers of inflammation, whereas NfL has emerged as a highly sensitive marker of neuroaxonal damage. IL-6 is a pleiotropic cytokine with both peripheral and central effects that is capable of crossing the blood–brain barrier (BBB), influencing neuronal function. Epidemiological studies have linked elevated IL-6 (and, to a lesser extent, CRP) levels in middle-aged individuals to later cognitive decline [36]. For instance, in the Whitehall II cohort, higher IL-6 (but not CRP) predicted a 10-year decline in reasoning, roughly corresponding to an age effect of nearly four years [37]. Meta-analyses further corroborated the association of inflammatory markers with neurodegenerative conditions: a recent meta-analysis revealed that IL-6 (and to a lesser degree, CRP) is elevated in AD and MCI patients [38].

In a longitudinal study of 138 cognitively normal older adults from the Baltimore Longitudinal Study of Aging, higher baseline and average levels of CRP and IL-6 over approximately five years were associated with greater declines in regional cerebral blood flow, an index of brain function, particularly in regions critical for memory and executive processes, including the hippocampus, medial temporal cortex, anterior cingulate, medial and orbitofrontal cortex, temporal cortex, putamen, and brainstem. These associations largely persisted after accounting for brain atrophy and white matter lesions, indicating functional vulnerability beyond structural damage. Higher CRP levels were also linked to poorer attention at baseline and greater longitudinal decline in visuospatial performance, although broader cognitive effects were limited. Therefore, systemic inflammation in aging was associated with progressive reductions in brain activity in regions known to be vulnerable in preclinical neurodegeneration, potentially increasing susceptibility to future cognitive decline and dementia [39].

A substantial body of evidence now indicates that chronic inflammation contributes to neurodegeneration, which occurs primarily via microglial activation, synaptic dysfunction, and possibly vascular injury (reviewed by Antwi et al.) [40]. The main mechanism involves excessive production of IL-6 produced by astrocytes or infiltrating immune cells, which promotes a proinflammatory milieu in the central nervous system (CNS) and elevates systemic IL-6, which then stimulates central immune activation. Chronic, age-related inflammation plays an important role in brain aging and contributes to cognitive decline even in the absence of overt disease. IL-6 and CRP normally help regulate neuronal processes involved in learning and memory but become chronically elevated with age and in conditions such as MCI and AD. Elevated CRP has also been found in the brains of AD patients (e.g., in plaques), suggesting that its peripheral elevation may reflect or even contribute to local pathological processes [40]. Moreover, in Parkinson’s disease (PD), data from over 150 studies (including 9032 patients diagnosed with PD and 12,628 controls, with 92 markers analysed) revealed that IL-6 and CRP were consistently higher in patients than in controls, as reviewed in a meta-analysis [41].

### 5.2. Critical Updates on mCRP as a Driver of AD

One of the earliest mechanistic links was provided by Strang et al., who demonstrated that β-amyloid (Aβ) plaques are capable of dissociating native pentameric CRP (pCRP) into the highly proinflammatory monomeric isoform, thereby creating a localized inflammatory amplification system within AD tissue [42]. Importantly, mCRP, but not pCRP, was significantly enriched within the frontal cortex of AD brains, strongly suggesting that local conversion of CRP occurred directly within amyloid-rich regions. Slevin et al. subsequently showed that direct hippocampal injection of mCRP into mice induced behavioural and cognitive deficits together with abnormal neuronal morphology, tau phosphorylation, amyloid-β accumulation, vascular permeability changes, angiogenesis, and activation of the ERK1/2 and IRS-1 signalling pathways associated with AD pathology [43]. Furthermore, mCRP colocalized with Aβ plaques, *p*-tau-positive neurons, and abnormal cerebral microvessels in human AD and ischemic stroke tissue, supporting the hypothesis that mCRP may mechanistically link cerebrovascular injury to accelerated dementia progression. More recently, Zhang et al. demonstrated that mCRP interacted directly with endothelial CD31 in an ApoE genotype-dependent manner, particularly within ApoE4 models, producing cerebrovascular inflammation, endothelial dysfunction, T lymphocyte extravasation, oxidative stress pathway activation, and impaired vasculogenic signalling, thereby establishing an ApoE4–mCRP–CD31 axis as a potential mediator linking peripheral inflammation to AD risk [44]. Consistent with these findings, Gan et al. reported that mCRP directly induced cellular AD pathology in primary neurons, once again increasing amyloid-β production, tau phosphorylation, neuronal apoptosis, and neurodegenerative biomarker expression in an ApoE-dependent pattern of ApoE4 > ApoE3 > ApoE2, whereas pentameric CRP had minimal effects [45]. Importantly, these effects occurred predominantly in neurons rather than in glial cells, suggesting that mCRP may directly trigger neuronal degenerative pathways independent of classical inflammatory gliosis. Finally, Na et al. [46] further demonstrated that peripheral mCRP disrupted protective ApoE-LRP1 interactions, concomitantly reducing pericyte-associated vascular stability while suppressing synaptic proteins, including synaptophysin and PSD95, and enhancing neuroinflammatory and tauopathic signalling during chronic inflammation. These findings further support a direct role for mCRP in neurovascular degeneration and AD progression [47]. These studies strongly support the concept that mCRP is not merely an inflammatory biomarker associated with AD but rather an active pathogenic mediator capable of driving amyloid-associated inflammation, endothelial dysfunction, BBB disruption, tauopathy, neuronal injury, and neurovascular degeneration, particularly in genetically susceptible [ApoE4] individuals.

### 5.3. NfL as a CRP-Equivalent Biomarker of Inflammation in the Brain

While inflammatory markers reflect immune activation, NfL provides a more direct readout of neuronal injury. Neurofilaments are structural cytoskeletal proteins that are particularly abundant in the axons of large-calibre neurons; when axons are damaged, NfL is released into extracellular spaces, CSF, and ultimately blood [48]. Due to ultrasensitive assays (e.g., Simoa), NfL can now be reliably quantified in serum or plasma, making it accessible for clinical and research use. Elevated NfL has been observed across a wide spectrum of neurological diseases, such as AD, PD, multiple sclerosis, and amyotrophic lateral sclerosis, to quantify tissue damage rather than to indicate pathogenic potential or predict outcome [49].

Findings from a comprehensive study on various neurodegenerative diseases (including almost all prion disease subtypes and variants of AD, dementia with Lewy bodies, and frontotemporal lobar degeneration) provided strong evidence that NfL is a robust discriminatory biomarker of neuronal damage. For example, some findings have shown that while NfL elevation is universal in these conditions, its levels and correlation with total-tau (t-tau) differ significantly among subtypes, indicating that NfL and t-tau reflect distinct pathological processes: t-tau is linked mainly to neuronal (gray matter) degeneration, whereas NfL is strongly influenced by subcortical axonal (white matter) pathology. Specifically, the prion disease subtypes with prominent subcortical involvement presented the highest NfL concentrations, often despite relatively low t-tau. NfL has proven valuable as a rapid screening tool, supporting its role in the differential diagnosis of rapidly progressive neurodegenerative disorders [50].

Given its sensitivity and broad applicability, researchers have sometimes called NfL a “neurological CRP”, although this term could be misleading” because it provides a universal signal of tissue damage in the nervous system, unlike CRP, which reflects systemic inflammation (and may fluctuate with infections or comorbidities), NfL more specifically mirrors neuroaxonal injury. The combination of inflammatory markers (CRP, IL-6) and NfL could thus provide a complementary biomarker framework: inflammation may contribute to or precede injury, whereas NfL captures accumulating neuronal damage [51]. NfL was a key indicator of neurodegeneration in a study by Elahi et al., with particularly pronounced relevance in early-onset Alzheimer’s disease (EOAD). Plasma NfL levels were significantly elevated in EOAD patients compared with controls after age adjustment, whereas increases in late-onset AD (LOAD) were weaker and did not consistently reach significance, highlighting more aggressive axonal damage in EOAD patients. NfL also loaded strongly onto the “degenerative” principal component alongside glial fibrillary acidic protein and was associated with greater white matter hyperintensity burden, linking axonal injury to cerebrovascular pathology. Although NfL has more modest associations with cognition than do trophic factors, its elevation, especially in EOAD, supports its utility as a sensitive plasma biomarker of disease intensity and neuroaxonal degeneration in AD [52].

Importantly, the interpretation of CRP isoforms and NfL is highly context dependent and may vary according to disease stage, inflammatory burden, degree of tissue injury, and therapeutic status. During acute inflammatory conditions, such as severe infection, active autoimmune flares, or critical illness, CRP and NfL often increase in parallel, reflecting simultaneous systemic inflammation and neuroaxonal injury. In contrast, in chronic neurodegenerative or mixed-pathology conditions, including aging, MCI, and AD, their relationship becomes less consistent, as NfL primarily reflects cumulative neuronal damage, whereas CRP more variably reflects the ongoing systemic inflammatory tone. Similarly, the biological interpretation of the pCRP/mCRP or mCRP:pCRP ratio likely differs according to the inflammatory context; high pCRP relative to mCRP may characterize acute-phase systemic responses, whereas increased relative mCRP may indicate sustained local tissue inflammation, endothelial activation, and chronic neurovascular injury. Importantly, most current studies have demonstrated associations rather than direct causality, and although mCRP appears to be mechanistically linked to endothelial dysfunction, microglial activation, oxidative stress, and BBB disruption, elevated NfL largely reflects downstream neuroaxonal injury rather than acting as a direct pathogenic mediator itself. Therefore, CRP isoforms and NfL should be interpreted as dynamic, complementary biomarkers reflecting different stages and components of the inflammatory–degenerative cascade rather than as isolated or universally interchangeable indicators.

In the pathological process of neurocognitive decline, individuals with RA with chronically active systemic inflammatory activity tend to develop lacunar stroke, which is strongly associated with the later development of AD, as illustrated in the section below.

### 5.4. Lacunar Strokes Are More Prevalent in Patients with RA and Could Be a Prognostic Predictor for AD

**Figure 2:**
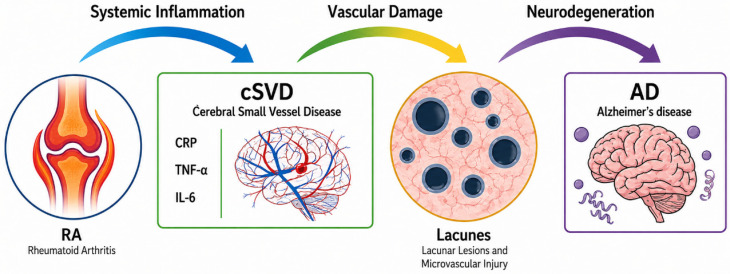
Proposed pathophysiological cascade linking rheumatoid arthritis (RA) to the development of Alzheimer’s disease (AD). Systemic inflammation in rheumatoid arthritis (RA), mediated by inflammatory biomarkers including C-reactive protein (CRP), tumour necrosis factor-alpha (TNF-α), and interleukin-6 (IL-6), may contribute to vascular inflammation, endothelial dysfunction, and the development of cerebral small vessel disease (cSVD) [53]. Chronic vascular injury and endothelial dysfunction may promote the formation of lacunar lesions (lacunes), impaired cerebral perfusion, and disruption of neurovascular integrity. Together these processes are hypothesized to contribute to neurodegeneration and cognitive decline and may potentially increase susceptibility to Alzheimer’s disease (AD) [57]. The figure represents a conceptual framework based on currently available associative evidence rather than an established causal pathway (generated by Google Gemini 3 Pro).

Lacunar infarcts are small (<15 mm), subcortical ischemic lesions that arise due to occlusion of a single penetrator (small artery) and are often secondary to lipohyalinosis or fibrinoid arteriolosclerosis. These lesions, along with white matter hyperintensities and microbleeds, constitute the imaging hallmarks of cerebral small vessel disease (cSVD). Clinically, lacunes may be “silent” (not producing overt stroke symptoms) but contribute significantly to cognitive decline, gait disturbances, and mood changes. Strategic lacunes (e.g., in the thalamus, internal capsule, or basal ganglia) can disproportionately impair cognition [53]. In a large memory-clinic cohort study (Amsterdam Dementia Cohort), the presence of lacunes on MRI was associated with alterations in CSF biomarkers of AD, particularly lower CSF tau in AD patients, indicating complex cross-talk between vascular and neurodegenerative processes. This association may be mediated or modified by genetic risk: in that same study, associations between vascular lesions and CSF biomarkers were more pronounced in APOE ε4 carriers, suggesting that genetic susceptibility modulates how vascular damage influences AD-type pathology [54]. Chronic inflammation in RA patients with elevated levels of CRP, IL-6, TNF-α, and other mediators drives endothelial activation and dysfunction, contributing to a prothrombotic, pro-atherogenic, and oxidative backdrop, resulting in increased vulnerability of small vessels (see Figure 2). Several pathological models of cSVD involve BBB disruption, perivascular inflammation, and remodelling of small arterioles [53].

The intersection of RA with cerebrovascular pathology, specifically cSVD and lacunar infarcts, contributes to neurodegenerative processes that may play a role in the development of AD. Lacunar strokes in RA potentially increase the risk of AD, with RA-driven vascular injury lowering the threshold for clinical AD manifestation by damaging microvascular networks, compromising brain structure and resilience, and interacting with AD pathology. Several epidemiological studies have shown that patients with RA have a greater incidence of dementia than individuals without RA. A large population-based cohort revealed that RA was associated with an increased risk of incident dementia, particularly in the presence of CV disease and stroke. For example, a Medicare-based study of 56,567 RA patients aged 65 years and over reported that those with CV disease and traditional vascular risk factors had significantly greater dementia risk (HR 1.18, 95% CI 1.04–1.33) [55]. Similarly, another cohort in North America investigated risk factors for incident dementia in patients with RA and reported that clinically active RA (specifically involving large joint swelling and rheumatoid nodules), along with established risk factors, was associated with increased dementia risk. CV disease, including hypertension and the occurrence of CV events (most strongly ischemic stroke and heart failure), significantly contributed to dementia risk in this population. Furthermore, depression and anxiety were also associated with incident dementia. Notably, single inflammatory markers, seropositivity (RF/ACPA), erosions, and disease-modifying antirheumatic drug (DMARD) use were not associated with dementia risk in this specific analysis. The study defined a high-risk phenotype for dementia in RA patients as an elderly individual with high RA disease activity, an unfavourable CV risk profile, and/or a mood disorder [56].

A nationwide Swedish registry-based cohort study (1685 patients with RA and 5055 patients without RA, followed for a median of approximately 2.8 years) that used repeated Mini-Mental State Examination (MMSE) scores and Cox proportional hazards models for all-cause mortality revealed that RA was independently associated with a significantly faster rate of cognitive decline (−0.24 MMSE points per year) and a higher risk of all-cause mortality (HR 1.15) than non-RA dementia patients were. These associations were consistent across multiple sensitivity and subgroup analyses, suggesting that chronic systemic inflammation contributes to accelerated cognitive decline and poorer survival in incident dementia patients [57].

Direct neuroimaging studies in RA are limited, but newer data support an elevated burden of subclinical cerebrovascular pathology. In the *Mayo Clinic Study of Aging*, RA participants presented more abnormalities in cerebrovascular imaging biomarkers, with more markers of cSVD (white-matter hyperintensities, silent infarcts) on MRI than did matched non-RA controls, despite no major differences in APOE ε4 status or neurodegenerative biomarkers [58]. A small but informative autopsy study compared cerebrovascular pathology in individuals with and without RA. While gross neuropathological differences were modest, RA patients presented more prominent cSVD, particularly in the basal ganglia. These regions are vulnerable to lacunar infarcts via small penetrating arteries, indicating a subclinical burden of cSVD in RA, even in the absence of overt dementia [59]. Further studies are needed to confirm whether some individuals with RA or other autoimmune diseases, including inflammatory bowel disease, are at greater risk of developing vascular damage within the brain or lacunar stroke pathology and whether this damage is associated with later onset of AD [60].

### 5.5. CRP Expression Is Correlated with cSVD

The *Rotterdam Scan Study* examined 1033 elderly adults without dementia and revealed that higher CRP levels predict both prevalent and incident white matter hyperintensities and lacunar infarcts. CRP is a marker for arteriolosclerosis and is actively involved in endothelial cell activation, with inflammation being a response to ischemic tissue damage. Higher CRP levels were associated with significantly higher white matter lesion scores, and the association remained after adjusting for CV risk factors. Over approximately 3 years, MRI scans revealed that 27% of patients developed progression of periventricular lesions and that 32% of patients developed progression of subcortical lesions. These data suggest that higher CRP levels may significantly accelerate the progression of cSVD in elderly people [61]. RA-driven systemic inflammation may accelerate cSVD development, leading to lacunar infarcts.

The relationships among systemic inflammation, cSVD, neurodegeneration and amyloid-related vascular pathology were examined in a population-based prospective cohort study conducted among middle-aged and elderly residents within the *Rotterdam Study*, which included 2814 dementia-free participants from the third cohort who underwent brain MRI and whose plasma CRP measurements were available. Brain MRI scans were performed on a 1.5-T scanner to evaluate markers of cSVD (lacunes, white matter hyperintensities, cerebral microbleeds, and enlarged perivascular spaces), as well as measures of neurodegeneration (gray matter, white matter, and hippocampal volumes), which were quantified via standardized imaging protocols. Plasma CRP levels were measured via a high-sensitivity immunoassay, and plasma amyloid-β (Aβ1-38, Aβ1-40, and Aβ1-42) concentrations were quantified in a subsample of 736 participants. Detailed demographic, vascular, and lifestyle covariates, including blood pressure, cholesterol, diabetes, smoking, medication use, and carotid atherosclerosis markers, were collected. Individuals with prevalent dementia were excluded, resulting in a final MRI sample of 2814 participants after exclusions for incomplete scans, infarcts, and missing CRP data [62]. Plasma CRP was assessed via a high-reliability immunoassay, whereas plasma Aβ1-38, Aβ1-40, and Aβ1-42 levels were quantified in a subsample via ELISA. Higher circulating CRP levels were associated with a greater burden of multiple MRI markers of cSVD, including increased white matter hyperintensity volume, greater lacunar and enlarged perivascular space counts, and a greater prevalence of deep or infratentorial cerebral microbleeds, while showing an inverse association with strictly lobar microbleeds. Elevated CRP was also associated with reduced gray matter volume. In the Aβ subsample, higher CRP levels were specifically associated with increased Aβ1-38 concentrations but not Aβ1-42 concentrations. Moreover, significant interactions between CRP and Aβ isoforms were observed, indicating that associations between CRP and cSVD markers (particularly lacunes, enlarged perivascular spaces, and microbleeds) were stronger among individuals with higher Aβ levels. Therefore, inflammatory processes may act synergistically with amyloid-related vascular pathology to exacerbate microvascular brain damage [62].

Once lacunar infarcts or white-matter lesions accumulate, they can undermine brain structural connectivity, especially in subcortical-cortical networks. These changes reduce “cognitive reserve” or compensatory capacity. In individuals with latent AD pathology (Aβ accumulation, tau), the added burden of vascular injury precipitated earlier or more severe clinical dementia. Furthermore, microvascular damage impaired glymphatic clearance, disrupted amyloid clearance, and promoted neuroinflammation, potentially accelerating AD pathology. Therefore, vascular and AD-type pathologies do not merely cooccur but may biologically interact, with genetic risk (e.g., APOE ε4) potentially amplifying this interaction [54]. In addition to vascular injury, RA inflammation may have more direct effects on the brain. Persistent systemic inflammation can prime microglia, increase BBB permeability, and promote neuroinflammatory cascades. Although specific studies in RA are limited, animal models of stroke have shown that inflammatory mediators (e.g., IL-1 and COX-2) exacerbate BBB disruption and leukocyte infiltration after ischemia [63].

### 5.6. RA Treatment and Dementia Risk

A systematic review and meta-analysis of over 940,000 patients revealed that biological DMARDs (bDMARDs), specifically TNF inhibitors, were associated with a significant 24–30% reduction in the risk of incident dementia among patients with RA. The study identified a distinct hierarchy of efficacy among TNF inhibitors, with etanercept providing the strongest protective benefit (42% reduction), followed by adalimumab (35%) and infliximab (20%). These targeted therapies likely prevent cognitive decline by suppressing chronic systemic hyperactivation of the immune response; specifically, they decrease the levels of proinflammatory cytokines such as TNF-α and IL-6. These cytokines, which are often elevated in the CSF of dementia patients, drive the pathogenesis of AD by stimulating microglia to trigger neuroinflammatory responses, which in turn accelerate the accumulation of Aβ plaques and the hyperphosphorylation of tau proteins. TNF inhibitors generally do not cross the BBB, suggesting that they work by reducing systemic inflammation rather than acting directly inside the brain [64].

## 6. Neuroimmune and CS Mechanisms of Pain in RA

Pain in RA and other musculoskeletal diseases is increasingly recognized as a multifactorial process that extends beyond peripheral joint inflammation. While inflammatory mediators contribute to nociceptor activation, persistent pain frequently reflects neuroimmune interactions involving peripheral sensitization, the CS, and maladaptive neuroplasticity. Cytokine signalling, autoantibody–neuronal interactions, and glial activation can alter nociceptive processing within both the periphery and CNS, allowing pain to persist even when inflammatory activity is controlled. These mechanisms help explain the frequent discordance between objective inflammatory markers and patient-reported pain, highlighting the need to understand distinct pain phenotypes and their neuroimmune drivers, for which we suggest here that CRP is a key protagonist in rheumatic disease.

### 6.1. Neuroimmune Mechanisms Driving Pain Beyond Joint Inflammation

Inflammation in joints triggers cytokines (e.g., TNF-α, IL-6), prostaglandins, leukotrienes, and neurotrophins, which activate peripheral nociceptors, and despite controlled inflammation, persistent pain could stem from pain ultrasensitization, which has been confirmed in several studies that used questionnaires, QSTs, and neuroimaging, as reviewed by Sofath and Lambarth. Genetic factors may contribute to musculoskeletal pain, particularly variants in serotonergic and adrenergic pathways, with the modulation of certain pathways (e.g., COMT and catecholamine signalling) showing therapeutic promise. Key clinical questions include whether early aggressive inflammation control could prevent sensitization and whether sensitized neural networks could be reversed [65].

A significant reduction in joint swelling and secondary hyperalgesia during the acute phase of antigen-induced arthritis (AIA) was observed in SNS-gp130^−/−^ mice lacking neuronal gp130 compared with controls. IL-6 signalling enhances the depolarization-evoked release of CGRP, a potent vasodilator, thereby facilitating neurogenic inflammation. While histopathological scores for cellular infiltration remained similar between genotypes, the loss of neuronal gp130 disrupted the typical correlation between swelling and tissue damage and resulted in lower systemic levels of IL-2 and IL-6. Notably, lymphocytes from SNS-gp130^−/−^ mice showed increased release of IL-17 and IFNγ upon antigen-specific restimulation, indicating that neuronal IL-6 signalling also serves to modulate the systemic cellular immune response [66].

### 6.2. Inflammation-Independent and CS Mechanisms

In a mouse model of collagen antibody-induced arthritis (CAIA), mechanical hypersensitivity and reduced locomotion occurred days before any visual or molecular signs of arthritis. Mouse and human sensory neurons express Fc gamma receptors (FcγRs), which are specialized proteins that bind the “Fc” tail of antibodies and are specific binding receptors of CRP. Specifically, anti-collagen type II (anti-CII) antibodies form immune complexes with soluble collagen fragments that directly activate these receptors on nociceptive (pain-sensing) neurons. In laboratory experiments, these immune complexes triggered calcium influx, inwards electrical currents, and the release of the pain-associated neuropeptide CGRP in sensory neurons, with FcγRs being critical mediators of autoantibody-induced pain. Pain occurred only when these receptors were present on nonhematopoietic cells (like neurons) and when the antibodies were properly glycosylated to bind them. This discovery of a direct “molecular dialogue” between the adaptive immune system and the nervous system suggested that targeting neuronal FcγRs could provide a new therapeutic pathway for treating pain in autoimmune conditions, even when inflammation is clinically controlled [67].

Lampa et al. reported that RA patients presented markedly elevated levels of the proinflammatory cytokine IL-1β in their CSF, with concentrations nearly 50 times higher than those of healthy controls; these elevated central levels occurred despite an intact BBB [68]. Peripheral joint inflammation triggers the CNS’s own resident immune cells (microglia and astrocytes) to produce cytokines independently, linking central IL-1β levels with the systemic symptoms of RA, specifically fatigue and poor sleep quality. While joint pain and tenderness do not correlate directly with CSF cytokine levels, the severity of fatigue does, indicating central neuroimmune activation in RA. In animal models of arthritis in which recipient C57BL/6 mice were injected with pooled sera collected from arthritic K/BxN transgenic mice to induce inflammatory arthritis and monitored over an 18-day period, during which clinical arthritis scores were recorded on the basis of the degree of swelling in each paw, increased mRNA expression of IL-1β, IL-18, and TNF was detected in the spinal cord, coinciding with the peak of joint swelling. Peripheral inflammation induces central neuroinflammatory responses even in the absence of overt BBB disruption, and the observed effects result from indirect neuroimmune communication pathways, including prostaglandin-mediated signalling across the brain and spinal endothelial cells, activation of endothelial cytokine receptors, and afferent neural signalling. These mechanisms may subsequently contribute to microglial priming and central sensitization, particularly at the spinal cord level, the primary site of neuroinflammatory activation in this model [68].

### 6.3. CRP-Associated Neuroplasticity, Pain Phenotypes, and Clinical Implications

Murine intra-articular injection of Freund’s adjuvant (CFA) represents an appropriate model of knee osteoarthritis, showing hallmarks of human disease (chronic joint inflammation induces marked structural and cellular remodelling, including synovial inflammation, meniscal displacement, macrophage infiltration (CD68+), and aberrant neovascularization (CD31+)). Using this model, Ghilardi et al. reported that CFA-induced arthritic joints exhibited robust sprouting of sensory (CGRP+, NF200+, GAP43+) and sympathetic (TH+) nerve fibres into regions of the synovium and periarticular tissue that are normally sparsely innervated, with these fibres displaying disorganized morphology and increased density [69]. Behavioural assessments revealed consistent persistent pain-related phenotypes, including increased spontaneous flinching, decreased limb use during ambulation, impaired rotarod performance, and reduced weight bearing on the affected limb. Notably, systemic administration of an anti-nerve growth factor (NGF) antibody (mAb 911) early in and throughout the disease course effectively prevented sensory and sympathetic nerve fibre sprouting and significantly attenuated all measured pain behaviours by up to 80% for spontaneous pain and 75% for dynamic weight bearing without altering macrophage infiltration or pathological neovascularization. These findings demonstrated that NGF-mediated activation and sprouting of TrkA+ nerve fibres, rather than structural joint damage or inflammation per se, are critical for chronic arthritic pain, with the NGF/TrkA signalling pathway as a potential key therapeutic target for modulating skeletal pain independently of inflammatory or vascular pathology [69].

**Figure 3:**
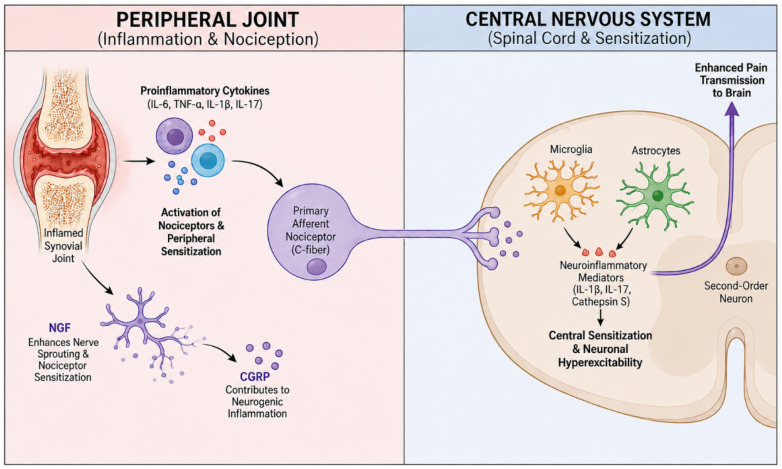
Mechanisms linking peripheral inflammation to central sensitization (CS) and pain in rheumatoid arthritis (RA). Peripheral joint inflammation promotes the release of proinflammatory cytokines, including IL-6, TNF-α, IL-1β, and IL-17, which activate primary afferent nociceptors (C-fibres) and contribute to peripheral sensitization. Nerve growth factor (NGF) enhances nerve sprouting and nociceptor sensitization, whereas calcitonin gene-related peptide (CGRP) contributes to neurogenic inflammation. Persistent nociceptive signaling from the inflamed synovium propagates to the spinal cord, where microglia and astrocytes amplify neuroinflammatory responses through mediators such as IL-1β, IL-17, and cathepsin S, promoting central sensitization and neuronal hyperexcitability. These processes collectively facilitate enhanced pain transmission to the brain and contribute to inflammation-independent chronic pain states in rheumatoid arthritis patients [70] (Generated by Google Gemini 3 Pro).

Consistent with the neurovascular remodelling observed in experimental arthritis models, studies of human joint tissues have similarly demonstrated increased vascularization and nerve growth within osteochondral structures. Using histomorphometric and immunohistochemical analyses of tibial plateau samples from patients with RA or OA and post-mortem controls, osteochondral angiogenesis and sensory nerve growth were characterized. Fibrovascular replacement of subchondral marrow, which is more pronounced in RA, is associated with osteochondral vascularization, suggesting a pathological microenvironment that promotes angiogenesis independently of synovitis (see Figure 3). VEGF and PDGF are expressed by subchondral cells and chondrocytes, with different patterns between RA and OA, whereas NGF is colocalized with CGRP-positive sensory nerve fibres within vascular channels, linking osteochondral angiogenesis to potential nerve sensitization [70].

Together, these findings suggest that peripheral joint pathology and neurovascular remodelling contribute to nociceptive signalling in arthritis; however, they do not fully explain the persistence and variability of pain experienced by patients. A 2021 study introduced the Central Aspects of Pain (CAP)-RA questionnaire as a means of assessing central pain mechanisms in RA and noted that many patients experienced substantial pain and fatigue even when inflammatory disease activity was well controlled. These findings suggest that CS contributes to ongoing symptoms and could inflate patient-reported components of disease activity indices, such as tender joint counts and visual analogue scales, even in the presence of low ESR or CRP levels [71].

Building on these observations, subsequent studies have sought to quantify central sensitization in RA patients via standardized neurophysiological measures. The Central Pain in Rheumatoid Arthritis (CPIRA) study investigated the contribution of central pain mechanisms to patient-reported pain in 263 patients with active RA initiating or changing DMARD therapy. Central pain mechanisms, including pressure pain thresholds (PPTs) at the trapezius to evaluate extra-articular pain sensitivity, temporal summation (TS) to measure spinal hyperexcitability, and conditioned pain modulation (CPM) to assess descending analgesic pathways, were assessed via quantitative sensory testing (QST). Pain intensity and interference were measured via PROMIS questionnaires, and analyses were adjusted for demographics, disease activity, seropositivity, inflammatory markers, and comorbidities. The study revealed that lower PPTs and higher TSs, indicative of central dysregulation, were significantly associated with greater pain intensity independent of inflammation, whereas CPM showed no consistent association, indicating alternative mediators of sensitivity and ultimately ‘chronic risk of disease’ predictors for which we propose CRP [72].

Over the course of 2 years, pain DETECT questionnaires were given to 180 RA patients to assess the non-nociceptive pain phenotype; individuals with RA experienced higher pain scores, greater disability, and lower physical and mental quality of life than patients with nociceptive pain did. Non-nociceptive pain was strongly associated with comorbid fibromyalgia and pain-related conditions. Despite similar objective markers of inflammation (swollen joint count and ESR), non-nociceptive patients consistently had higher DAS28 scores over time, driven mainly by subjective measures such as tender joint count and patient global health assessment, and were less likely to achieve sustained remission, with pain in RA resulting from CS rather than inflammation alone [73].

Using nationally representative data from the U.S. National Health and Nutrition Examination Survey (NHANES) from 1999–2004, a cross-sectional study investigated the association between CRP levels and chronic pain in 10,680 adults aged 20 years and over. Chronic pain was defined according to the International Classification of Disease-11 (ICD-11) criteria and assessed via self-reported pain questionnaires. Approximately one quarter of the participants reported chronic pain. Individuals with chronic pain were more likely to be female, non-Hispanic White, obese, of lower socioeconomic status, current smokers, and to have multiple comorbidities (e.g., diabetes, cardiovascular disease, lung disease, and cancer). These patients also had higher levels of inflammatory markers, including CRP. Higher CRP levels were significantly associated with a greater likelihood of chronic pain. After full adjustment for confounders, participants in the highest CRP quartile had 32% greater odds of chronic pain than those in the lowest quartile did. A significant linear dose–response relationship between CRP levels and chronic pain risk was observed, supporting a meaningful association between systemic inflammation and chronic pain and suggesting that inflammatory pathways contribute to pain persistence [74].

### 6.4. Individualized Mechanisms of Pain Operating Through CRP: Biological, Psychological, and Social Factors in Chronic Pain Sensitivity and Inflammation

A study by Lee et al. investigated pain sensitivity in patients with RA and reported that pain persisted due to both inflammatory and noninflammatory mechanisms. In 59 female RA patients, pressure pain thresholds were measured at joints, near joints, and distant non-joint sites and analysed alongside markers of inflammation (CRP), sleep problems, and psychiatric distress. After adjustment for confounders, higher CRP levels were independently associated with lower pain thresholds at the wrists (and borderline at nearby sites) but not at distant sites, supporting a role for peripheral sensitization driven by local inflammation. In contrast, sleep problems were strongly associated with lower pain thresholds at all sites, indicating a central pain processing mechanism underlying widespread pain sensitivity. Psychiatric distress was related to pain sensitivity in unadjusted analyses but not after accounting for sleep, reflecting their strong interrelationship. Although patients with fibromyalgia-like features presented greater pain sensitivity and sleep disturbance, the main findings persisted after excluding them. Therefore, RA pain sensitivity reflects a combination of local inflammatory effects and central factors, particularly sleep disturbance [75].

Similarly, a large cross-sectional study used baseline data from the UK Biobank (>415,000 participants aged 40–69 years) to examine associations between blood CRP and chronic pain across multiple body sites while rigorously adjusting for a comprehensive set of demographic, clinical, psychological, and lifestyle variables and stratifying analyses by sex. CRP levels were consistently higher in individuals with chronic pain than in pain-free controls for all pain sites in both females and males, with the highest levels observed in widespread pain; low-grade inflammation (CRP 3–10 mg/L) was present in approximately 19–24% of those with chronic pain compared with 14–17% of controls, and markedly elevated CRP (>10 mg/L) was particularly common in widespread pain. Although adjustment for biopsychosocial confounders attenuated effect sizes, higher CRP remained significantly associated with all pain types in males and most pain types in females, with the strongest and most consistent associations observed for widespread pain, whereas associations with facial pain and headache were no longer significant in females [76].

Kato et al. investigated whether objective pain sensitivity measures could help characterize psychological traits in patients with chronic pain. Fifty-seven adults with nonmalignant chronic pain were recruited, and pain tolerance thresholds (PTTs) were measured at noninjured sites via QST with electrical stimulation targeting Aδ and C fibres, an approach intended to reflect the CS rather than local tissue pathology. The participants also completed standardized assessments of pain intensity and the Minnesota Multiphasic Personality Inventory (MMPI) to evaluate personality and psychological traits. Using cluster analysis of PTTs, the patients were classified into a ‘high-sensitivity group’ with markedly reduced pain tolerance and an ‘others’ group with higher and more variable thresholds. Demographic factors, clinical pain measures, and MMPI profiles were compared between the groups. Despite similar self-reported pain severity and duration, the high-sensitivity group presented distinct psychological characteristics, including a higher prevalence of the Neurotic Triad MMPI pattern and lower hypochondriasis and hysteria scores, with no cases of the Conversion V pattern. Classifying chronic pain patients on the basis of pain tolerance thresholds at noninjured sites revealed meaningful psychological differences not captured by subjective pain reports [77].

Pain perception varies widely due to interactions among biological, psychological, and social factors. Genetic variants, demographic characteristics, and psychosocial factors such as stress and catastrophizing all influence pain sensitivity. In RA, pain results from both inflammatory processes and central mechanisms that increase widespread pain sensitivity. The combined role of inflammation and CS in pain experiences has been proven through large population studies, which have shown that elevated CRP is associated with chronic pain.

In summary, accumulating evidence increasingly links CRP, the CRP gene, and particularly mCRP, not only to systemic inflammation in RA but also to altered nociceptive processing, stress hypersensitization, vascular injury, and subsequent neurodegenerative risk. As discussed throughout the manuscript, persistent CRP elevation in RA correlates more consistently with endothelial dysfunction, cerebral small vessel disease (cSVD), and chronic inflammatory burden than many traditional inflammatory markers do, while emerging evidence suggests that mCRP may actively participate in neurovascular and neuroimmune signalling pathways involved in pain sensitization and tissue injury. Chronic pain in RA is increasingly recognized as a manifestation of central sensitization and maladaptive neuroimmune activation rather than simply joint inflammation alone, with CRP-associated inflammatory signalling potentially contributing to sustained sympathetic activation, microglial priming, BBB dysfunction, and progressive vascular damage. These processes are highly relevant to cognitive decline and AD risk because vascular inflammation, lacunar injury, impaired cerebral perfusion, and chronic neuroinflammation are now considered important mechanistic contributors to accelerated neurodegeneration in RA. Accordingly, we believe that CRP may function not only as a biomarker of inflammation but also as an integrated indicator of chronic stress-associated neurovascular injury, linking RA disease activity to increased long-term dementia and AD susceptibility [78].

## 7. Therapeutic Implications and Future Directions

Future therapeutic approaches are likely to focus on two complementary strategies: indirect reduction in mCRP generation through good control of systemic and local inflammation and direct inhibition of mCRP formation or activity. Optimized treat-to-target RA management via conventional synthetic DMARDs, biologics and targeted synthetic DMARDs may limit the conversion of pCRP to mCRP by reducing the inflammatory microenvironment. In addition, preclinical studies have demonstrated that monoclonal antibodies, aptamers and small molecules capable of neutralizing mCRP or preventing pCRP dissociation can attenuate vascular inflammation and arthritis severity in animal models [79]. Dissociation into mCRP at sites of tissue injury occurs when pCRP binds phosphocholine residues exposed on cell membranes via phospholipase A2 (PLA2) enzymes, generating an intermediate form that ultimately separates into mCRP, particularly following complement C1q binding. Therapeutic strategies could include indirect inhibition through PLA2 inhibitors (targeting sPLA2, cPLA2, and LpPLA2) and direct inhibition of CRP dissociation. However, major PLA2 inhibitors, such as darapladib and varespladib, have failed in late-stage clinical trials, and the only direct CRP dissociation inhibitor developed, 1,6-bis(phosphocholine)-hexane, has demonstrated limited pharmacokinetic viability despite success in animal models [80]. While these data are compelling, direct mCRP-targeting therapies remain investigational, and human randomized clinical trials are still needed before routine clinical use can be recommended [81].

As a biomarker, mCRP may offer potential advantages over conventional inflammatory markers by reflecting localized tissue-level inflammation rather than systemic acute-phase responses alone [18]. Recent observational studies have indicated that elevated circulating mCRP is associated with increased CV risk independent of hsCRP and traditional risk factors. In RA, incorporation of mCRP into prognostic models could improve identification of patients at heightened risk for CV complications, progressive joint damage or extra-articular manifestations. In addition, mCRP may be particularly valuable for reclassifying patients with intermediate-risk profiles or persistent inflammation despite apparently controlled disease according to standard clinical metrics [82]. Furthermore, mCRP has potential as a complementary biomarker for assessing residual inflammatory CV risk and improving early detection of aggressive coronary artery disease (CAD) in younger populations. While conventional hsCRP reflects systemic inflammation and residual inflammatory CV risk, mCRP is a tissue-bound, proinflammatory form generated through the dissociation of pCRP at sites of cellular damage. In a case–control study of 103 participants (50 with premature CAD and 53 controls), patients with CAD presented significantly higher plasma mCRP levels than did those without CAD (median 6.84 vs. 2.57 µg/L, *p* < 0.001), whereas hsCRP levels were not significantly different. Notably, among participants with hsCRP < 2.0 mg/L (typically considered low risk), mCRP levels remained elevated in the CAD group. Multivariate logistic regression, adjusted for traditional risk factors, demonstrated that mCRP was independently associated with premature CAD (odds ratio 1.18 per µg/L increase, 95% CI 1.06–1.33, *p* = 0.004). ROC analysis indicated an optimal mCRP cut-off of 4.41 µg/L, providing 74% sensitivity and 69.8% specificity for CAD classification [79].

Wang et al. identified mCRP as a key therapeutic target in RA and OA and demonstrated that selective neutralization of mCRP effectively suppressed disease progression. In hepatocyte-specific CRP knockout mice, liver-derived CRP was shown to be a causal driver of joint inflammation, synovial hyperplasia, cartilage destruction, and bone remodelling in multiple RA and OA models. mCRP induced pathogenic activation of fibroblast-like synoviocytes, promoted proinflammatory M1 macrophage polarization, and drove chondrocyte catabolism through FcγRI-dependent signalling. To exploit this pathway therapeutically, a highly specific single-stranded DNA aptamer, ApmCRP3, which selectively binds and neutralizes mCRP without affecting pCRP, was designed. ApmCRP3 reversed mCRP-mediated inflammatory and degradative responses in vitro and significantly attenuated arthritis severity in both the RA and OA mouse models, outperforming the CRP-stabilizing agent 1,6-bisPC while showing no detectable toxicity. These findings establish mCRP as a pathogenic effector, suggesting strong proof-of-concept that aptamer-based targeting of mCRP represents a precise and promising therapeutic strategy for both inflammatory and degenerative arthritis [81].

**Figure 4:**
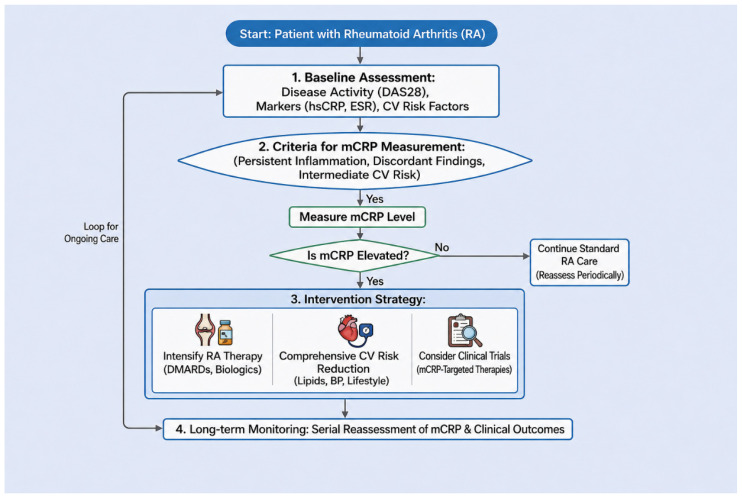
Proposed clinical workflow for the assessment and management of monomeric C-reactive protein (mCRP) in rheumatoid arthritis (RA) patients. Patients with RA undergo baseline evaluation, including disease activity assessment (DAS28), inflammatory marker (hsCRP, ESR), and cardiovascular (CV) risk factor profiling. The measurement of mCRP is considered useful in patients with persistent inflammation, discordant clinical and laboratory findings, or intermediate cardiovascular risk. Patients without elevated mCRP levels continue standard RA care with periodic reassessment. Elevated mCRP levels may prompt intensified RA therapy (including DMARDs or biologics), comprehensive cardiovascular risk reduction strategies, and consideration for enrolment in clinical trials targeting mCRP-related pathways [84]. Long-term follow-up includes serial reassessment of mCRP levels and clinical outcomes as part of ongoing patient management. (Generated by Google Gemini 3 Pro).

A practical algorithm that integrates mCRP into RA management may involve the following steps. First, baseline assessments of RA disease activity (e.g., DAS28), traditional inflammatory markers (hsCRP, ESR) and CV risk factors are performed. Second, mCRP should be measured in patients with persistent inflammatory activity, discordant clinical and laboratory findings, or intermediate CV risk [83]. Third, patients with elevated mCRP undergo intensified RA therapy and comprehensive CV risk reduction strategies, including lipid-lowering therapy, blood pressure optimization and lifestyle interventions. Where available, enrolment in clinical trials investigating mCRP-targeted therapies may be considered. Finally, serial reassessment of mCRP alongside clinical outcomes would guide ongoing treatment decisions (see Figure 4) [84].

On the basis of existing RA treat-to-target paradigms, reassessment of disease activity and biomarkers should occur ~3 months after therapy initiation or escalation, with subsequent evaluations every 3–6 months until stability is achieved. For patients identified as high-risk on the basis of elevated mCRP, repeated mCRP measurement at 3 months could provide early insight into biological response, with longer-term monitoring at 6–12 months to assess durability of risk reduction [83]. Imaging modalities such as carotid ultrasound or coronary CT may be considered for select patients with persistently elevated mCRP to detect subclinical vascular disease. Successful risk reduction is reflected by sustained decreases in mCRP, improved RA disease control and the stabilization of surrogate CV imaging markers [85].

Innovative therapeutic strategies for AD include the inhibition of the NLRP3 inflammasome, which represents a shift from modulating downstream effects to targeting a central upstream driver of AD pathogenesis. As a cytosolic multiprotein complex predominantly expressed in microglia, NLRP3 acts as a key sensor of cellular stress. In AD, it is directly activated by pathological protein aggregates, including β-amyloid and hyperphosphorylated tau. Once activated, NLRP3 initiates a self-reinforcing pathological cascade: the activation of caspase-1 promotes the maturation and release of the proinflammatory cytokines IL-1β and IL-18. These cytokines both induce pyroptosis (an inflammatory form of programmed cell death) and exacerbate the accumulation and spread of misfolded proteins. NLRP3 functions as a disease-amplifying focal point linking central neuroinflammation with systemic inflammatory responses. For example, IL-1β signalling extends beyond the central nervous system and into systemic circulation, where it stimulates hepatic acute-phase responses and increases the levels of CRP [86].

Peripheral blood monocytes from patients with MCI and AD were analysed to define the role of the NLRP3 inflammasome. While NLRP3 is present in MCI, it only successfully assembles into a fully active, cytokine-cleaving complex in the cells of patients who have progressed to AD. In this study, the cells were initially primed with LPS to induce protein production and then triggered with Aβ42 to simulate AD pathology. NLRP3 mRNA and the 75 kDa protein isoform were significantly upregulated in AD and MCI patients compared with healthy controls. NLRP3 physically colocalized with the adaptor protein PYCARD and caspase 1 in AD patients but not in MCI patients. Furthermore, FLICA fluorescent probes were used to confirm that NLRP3 actively signals through caspase 1 and caspase 8 in severe AD patients [87]. Targeting NLRP3 through small-molecule inhibitors or metabolic interventions represents a promising disease-modifying strategy. Such approaches aim to attenuate neuroinflammation simultaneously, reduce Aβ and tau pathology, and disrupt the feed-forward inflammatory loop. Although challenges remain, particularly with respect to BBB penetration and the risk of immunosuppression, NLRP3 inhibition remains one of the most compelling investigational avenues for reshaping the therapeutic landscape of AD.

The long-term effects of mCRP on cognitive function and neurodegeneration were evaluated in a mouse model (3-month-old male C57BL/6J mice) that received a single bilateral intrahippocampal injection of mCRP. Three independent studies lasting 1, 3, and 6 months were conducted to assess the progression of behavioural and pathological changes. To test potential therapeutic interventions, a specific monoclonal antibody (8C10) was administered to block mCRP, or an oral inhibitor of the epoxide hydrolase enzyme (TPPU) was provided. Cognitive assessments were performed via the novel object recognition test (NORT) and the Morris water maze (MWM), and brain tissues were analysed to detect tau phosphorylation and gene expression. The study revealed that a single exposure to mCRP induced persistent and total memory loss in both the recognition and spatial paradigms, which remained evident up to 6 months post-injection. Pathological analysis revealed that mCRP triggered significant tau hyperphosphorylation at the AT8 and Ser396 epitopes, mirroring hallmarks of AD. A decrease in the neural plasticity marker Egr1, similar to that observed in transgenic AD mice, was observed. In vitro assays using BV2 microglia demonstrated that mCRP, but not the native pentameric form, significantly increased nitric oxide generation and iNOS levels. In conclusion, the 8C10 antibody successfully prevented memory loss, blocked tau pathology, and inhibited microglial activation, whereas TPPU provided similar cognitive protection by mitigating the inflammatory response [88].

Future RA pain research should specifically address how chronic systemic inflammation induces long-term neuroimmune alterations within the peripheral nervous system and CNS and should integrate neuroimmune biomarkers with advanced pain phenotyping. Longitudinal studies combining DAS28, QST, functional MRI, pain catastrophizing assessment, sleep and fatigue evaluation, and biomarkers (IL-6, TNF-α, NfL, GFAP, CRP, and mCRP) could help identify patients at risk of developing persistent CS despite inflammatory remission. It will also be important to determine whether biologic therapies differentially modulate central pain pathways; for example, IL-6 inhibition may have stronger effects on fatigue, mood, and centrally mediated pain than therapies that primarily target peripheral inflammation alone [89].

Another important direction involves the development of mechanism-based therapeutic strategies specifically targeting neuroimmune interactions. These may include glial-modulating therapies, selective cytokine pathway inhibition, vagal nerve stimulation, neuromodulation techniques, cognitive behavioural interventions, and personalized rehabilitation programmes designed to reduce CS.

Despite its promise as a biomarker and potential therapeutic target, several critical barriers must be addressed before mCRP can be fully integrated into routine RA and AD care. First, there is a pressing need for standardized, clinically validated mCRP assays. Current detection methods vary in sensitivity, specificity, and reproducibility, making it challenging to compare results across studies or implement them in clinical practice. Alongside assay standardization, clinically meaningful cut-off values must be established to reliably distinguish between different disease activity states, predict flares, or guide therapy adjustments. Equally important are prospective, longitudinal studies that evaluate whether mCRP-guided management strategies actually translate into improved patient outcomes, which include reduced joint damage, enhanced quality of life, memory loss prevention, or reduced systemic inflammation. In addition to its role as a biomarker, mCRP represents a potential therapeutic target, yet evidence supporting direct mCRP inhibition in RA is currently limited. Well-designed randomized controlled trials are essential to determine whether interventions that specifically block mCRP can meaningfully reduce RA-associated comorbidities, including CV complications and neurodegeneration, beyond what is achievable with standard anti-inflammatory therapies and CV risk-modifying strategies. Addressing these gaps is essential to translating the biological insights around mCRP into tangible benefits for patients.

## Data Availability

No new data were created or analyzed in this study. Data sharing is not applicable to this article.

## Abbreviations

The following abbreviations are used in this manuscript:

^18^F-FDG PET	Fluorodeoxyglucose Positron Emission Tomography
Aβ	Amyloid-Beta
ACPAs	Anticitrullinated Peptide Antibodies
AD	Alzheimer’s Disease
ADMA	Asymmetric Dimethylarginine
Aix	Augmentation Index
AIA	Antigen-Induced Arthritis
AOSD	Adult-Onset Still’s Disease
APOE ε4	Apolipoprotein E Epsilon 4 Allele
BBB	Blood–Brain Barrier
bDMARDs	Biological Disease-Modifying Antirheumatic Drugs
CAD	Coronary Artery Disease
CAIA	Collagen Antibody-Induced Arthritis
CAP-RA	Central Aspects of Pain in Rheumatoid Arthritis
CFA	Complete Freund’s Adjuvant
CGRP	Calcitonin Gene-Related Peptide
cIMT	Carotid Intima Media Thickness
COMT	Catechol-O-Methyltransferase
CPIRA	Central Pain in Rheumatoid Arthritis Study
CPM	Conditioned Pain Modulation
CNS	Central Nervous System
CRP	C-Reactive Protein
CRP/mCRP ratio	C-Reactive Protein/Monomeric C-Reactive Protein Ratio
CS	Central Sensitization
CSF	Cerebrospinal Fluid
cSVD	Cerebral Small Vessel Disease
CX3CR1	C-X3-C Motif Chemokine Receptor 1
CXCL8	C-X-C Motif Chemokine Ligand 8
DAS28	Disease Activity Score in 28 Joints
DAS28-CRP	Disease Activity Score in 28 Joints Using CRP
DMARDs	Disease-Modifying Antirheumatic Drugs
EOAD	Early-Onset Alzheimer’s Disease
ERK1/2	Extracellular Signal-Regulated Kinase 1/2
ESR	Erythrocyte Sedimentation Rate
FcγRs	Fc Gamma Receptors
FLICA	Fluorescent-Labelled Inhibitor of Caspases
FLSs	Fibroblast-Like Synoviocytes
GFAP	Glial Fibrillary Acidic Protein
gp130	Glycoprotein 130
hsCRP	High-Sensitivity C-Reactive Protein
ICAM-1	Intercellular Adhesion Molecule 1
ICD-11	International Classification of Diseases, 11th Evision
IFNγ	Interferon Gamma
iNOS	Inducible Nitric Oxide Synthase
IL	Interleukin
IL-1Ra	Interleukin-1 Receptor Antagonist
JAK	Janus Kinase
JNK	C-Jun N-Terminal Kinase
LPC	Lysophosphatidylcholine
LPS	Lipopolysaccharide
LOAD	Late-Onset Alzheimer’s Disease
M-CSF	Macrophage Colony-Stimulating Factor
MAPK	Mitogen-Activated Protein Kinase
MCI	Mild Cognitive Impairment
mCRP	Monomeric C-Reactive Protein
MDA	Malondialdehyde
MI	Myocardial Infarction
MMPI	Minnesota Multiphasic Personality Inventory
MMSE	Mini-Mental State Examination
MMP9	Matrix Metalloproteinase 9
MPs	Microparticles
MRI	Magnetic Resonance Imaging
MWM	Morris Water Maze
NF-κB	Nuclear Factor Kappa B
NfL	Neurofilament Light Chain
NGF	Nerve Growth Factor
NHANE	National Health and Nutrition Examination Urvey
NLRP3	NOD-Like Receptor Family Pyrin Domain Containing 3
NORT	Novel Object Recognition Test
OA	Osteoarthritis
OPRM1	Opioid Receptor Mu 1
pCRP	Pentameric C-Reactive Protein
PD	Parkinson’s Disease
PDGF	Platelet-Derived Growth Factor
PI3K	Phosphoinositide 3-Kinase
PLA2	Phospholipase A2
PPT	Pressure Pain Threshold
PROMIS	Patient-Reported Outcomes Measurement nformation System
PWV	Pulse-Wave Velocity
PYCARD	PYD and CARD Domain-Containing Protein
QST	Quantitative Sensory Testing
RA	Rheumatoid Arthritis
RA-FLSs	Rheumatoid Arthritis Fibroblast-Like Synoviocytes
RANKL	Receptor Activator Of Nuclear Factor Kappa-B Ligand
RF	Rheumatoid Factor
scFv	Single-Chain Fragment Variable
sgp130Fc	Soluble Glycoprotein 130 Fc Fusion Protein
Simoa	Single-Molecule Array
TH	Tyrosine Hydroxylase
TLR	Toll-Like Receptor
TNF-α	Tumor Necrosis Factor-Alpha
TNFR1	Tumor Necrosis Factor Receptor 1
TNFR2	Tumor Necrosis Factor Receptor 2
TRAF	TNF Receptor-Associated Factor
TS	Temporal Summation
VCAM-1	Vascular Cell Adhesion Molecule 1
VEGF	Vascular Endothelial Growth Factor
